# Modulation of the Activity of Sp Transcription Factors by Mithramycin Analogues as a New Strategy for Treatment of Metastatic Prostate Cancer

**DOI:** 10.1371/journal.pone.0035130

**Published:** 2012-04-19

**Authors:** Anastasia Malek, Luz-Elena Núñez, Marco Magistri, Lara Brambilla, Sandra Jovic, Giuseppina M. Carbone, Francisco Morís, Carlo V. Catapano

**Affiliations:** 1 Institute of Oncology Research (IOR), Bellinzona, Switzerland; 2 Oncology Institute of Southern Switzerland (IOSI), Bellinzona, Switzerland; 3 EntreChem SL, Edificio Científico Tecnológico, Campus El Cristo, Oviedo, Spain; University of Hong Kong, Hong Kong

## Abstract

Deregulated activity of transcription factors (TFs) of the Sp/KLF family, like Sp1, Sp3 and Sp4, and consequent over-expression of Sp-regulated genes occur frequently in human cancers. This provides the rationale for development of inhibitors of Sp TFs as cancer therapeutics. Mithramycin A (MTM-A) is a natural polyketide that binds GC-rich DNA sequences, inhibits activity of Sp TFs and exhibits potent antitumor activity in experimental systems. However, clinical use of MTM-A is limited by the severe toxicity of the compound. Here, we studied two MTM-A analogues, which had been generated by genetically engineering of the MTM-A biosynthetic pathway, and evaluated their activity in human prostate cancer in cell cultures and mouse models. The compounds, named MTM-SDK and MTM-SK, were highly effective *in vitro* inhibiting proliferation of prostate cancer cells and transcription of Sp-regulated genes by blocking binding of Sp proteins to the gene promoters When administered to mice, both compounds were well tolerated with maximum tolerated doses of MTM-SDK and MTM-SK, respectively, 4- and 32- fold higher than MTM-A. After systemic administration, both compounds were cleared rapidly from the bloodstream but maintained plasma levels well above the active concentrations required *in vitro* for inhibition of Sp TF activity and cell proliferation. Consistently, MTM-SDK and MTM-SK inhibited transcription of Sp-regulated genes in prostate tumor xenografts and exhibited potent antitumor activity in subcutaneous and metastatic tumor xenograft models with no or minimal toxicity. Taken together, these data indicate that MTM-SDK and MTM-SK possess significantly improved pharmacological and toxicological properties compared to MTM-A and represent promising drugs for treatment of advanced prostate cancer.

## Introduction

Tumor initiation and progression are mediated by multiple signaling pathways and the therapeutic benefits achievable by targeting individual pathways may be limited [1]. Targeting the sites of convergence of diverse regulatory cascades may represent a more promising strategy for cancer treatment. Transcription factors (TFs) are particularly attractive in this regard since they are nodal points in signaling pathways and are frequently deregulated in human cancers [1], [2]. Aberrant expression or activity of the members of the Sp/KLF family of TFs, like Sp1, Sp3 and Sp4, occurs in many human cancers [3] Sp TFs bind to GC-rich DNA elements (GC-box) in gene promoters, interact with components of the basal transcriptional machinery, cooperate with other TFs and are downstream effectors of multiple signaling pathways [3]. Several studies have demonstrated that Sp TFs have important roles in the pathogenesis of human cancers and are promising therapeutic targets [3], [4], [5], .

Mithramycin A (MTM-A) is a natural polycyclic aromatic polyketide produced by various *Streptomyces* species [14]. MTM-A binds preferentially to GC-rich sequences in DNA, blocks competitively the binding of Sp TFs and other GC-binding proteins to GC-rich elements in gene promoters and inhibits transcription of Sp regulated genes [15], [16], [17], [18], [19]. Inhibition of Sp regulated genes is the major determinant of the biological activity of MTM-A in experimental systems [20], [21]. Clinical use of MTM-A however, is limited because of the toxicity of the compound [22]. Genetically engineering of the MTM-A biosynthetic pathway has given the opportunity to generate new analogues of MTM-A that might possess improved pharmacological and toxicological properties [23], [24], [25], [26]. Recently, two compounds, named MTM-SDK and MTM-SK, were obtained using the metabolic engineering approach [27], [28]. MTM-SDK and MTM-SK exhibited greater ability to block Sp1 binding to DNA and cellular uptake compared to MTM-A. This led to increased biological activity as inhibitors of Sp regulated gene transcription and proliferation of cancer cells both in vitro and in vivo [27], [29]. Thus, these new MTM-A analogues might be useful for treatment of cancers with abnormal activity of Sp TFs.

In this study, we evaluated the activity of the MTM-A analogues MTM-SDK and MTM-SK in experimental models of human prostate cancer. Prostate cancer is the most common cancer and the second leading cause of cancer death in men in western countries [30]. Conventional management of prostate cancer includes surgery, radiotherapy and androgen deprivation [31]. Despite the gradual increase in disease-free survival and quality of life, metastatic dissemination is still the main cause of death for prostate cancer patients [31]. Advanced prostate cancer is often resistant to hormonal treatment and systemic chemotherapy has limited efficacy [31], [32]. Palliative therapy remains the main option for many of these patients. Therefore, new therapeutic approaches need to be implemented to manage metastatic prostate cancer. The state of Sp TFs in prostate cancer has been poorly investigated to date. However, we found evidence supporting a role for deregulated activity of Sp TFs in initiation and progression of prostate cancer. Many genes reported to be involved in prostate tumorigenesis are regulated by Sp factors (see Methods S1; Table S1, S2 and references therein). Compounds that interfere with Sp TFs by different mechanisms have relevant activity in various cancers, including prostate cancer [7], [8], [9], [10], [11], [33], [34], [35], [36], [37], [38]. These considerations, along with bioinformatics analyses of published gene expression datasets, led us to hypothesize that Sp TFs could be relevant targets in prostate tumors and that the new MTM-A analogues with improved pharmacological properties might be useful for treatment of this disease.

## Materials and Methods

### Compounds and cell lines

Human prostate cancer PC3, DU145, 22Rv1 and LNCaP cells were maintained in RPMI 1640 [39]. Normal human fibroblasts were maintained in DMEM [27]. MTM-A, MTM-SK (EC-7072) and MTM-SDK (EC-7073) were prepared as previously described [27]. Stock solutions of the compounds (10 mM) were prepared in sterile saline solution or DMSO and diluted in sterile saline solution immediately before use [29]. For gene expression and chromatin immunoprecipitation (ChIP) cells were treated with 100 nM of MTM-SDK and MTM-SK or vehicle for 24 h.

### RNA and protein analysis

Total RNA from cultured cells and tumor tissues was isolated and reverse transcribed as described [27], [29]. Quantitative real-time RT-PCR (qRT-PCR) was performed using ABI PRISM 7000HT Sequence Detection System and SYBRGreen PCR Master Mix (Applied Biosystems) as described [29]. PCR primers are shown in Table S3. *GAPDH* and *B2M* were used as internal controls. Expression data were expressed as a percentage of control gene expression as described [29]. End point RT-PCR was performed as described previously using the SuperScript One-Step RT–PCR system (Invitrogen) and gene-specific primers [27]. Cell lysates were prepared from control and drug-treated cells and proteins separated on SDS–polyacrylamide gels. Immunoblotting was performed as described previously [27], [40].

### Chromatin immunoprecipitation (ChIP)

Cells were collected, cross-linked with formaldehyde and processed following a modified EZ-ChIP kit protocol (Upstate Biotechnology) as described [41]. Chromatin was immunoprecipitated with an antibody for Sp1 and normal mouse IgG as negative control. DNA-protein cross-links were reversed and DNA was purified from total cell lysates (input) and immunoprecipitated fractions. qPCR was performed as indicated above. Primers for ChIP analysis of the C-MYC and VEGF promoter are shown in Table S3. The amount of bound DNA was calculated in reference to a standard curve and expressed as percentage of input DNA [41].

### *In vitro* cell growth inhibition

Cell viability was determined using the MTT colorimetric assay as described [27]. Cells were plated in 96-well plates and incubated with vehicle or compounds for 24 and 72 h. Each treatment was performed in triplicate and experiments were repeated at least three times.

### Animals and xenograft models

Athymic male nude mice (Balb c nu/nu, 6–8 weeks old) were purchased from the Harlan Laboratories. Mice were maintained under pathogen-free conditions with food and water provided *ad libitum* and their general health status was monitored daily. All protocols involving laboratory animals were conducted in conformity with the institutional guidelines and in compliance with national and international laws and policies. Experimental procedures and study protocols have been reviewed and approved by the Swiss Cantonal Veterinary Authority (Approval no. 05/2011). To establish subcutaneous tumor xenografts, PC3 cells (2×10^6^ cells) were inoculated in the flank of mice. For the metastatic xenograft model, PC3 cells (0.6×10^6^) were injected twice into the tail vein with a 24-h interval between injections.

### Toxicity

Healthy CD-1 mice provided by the University of Oviedo Animal Facility were treated with single or repeated IV injections of MTM-SK, MTM-SDK and MTM-A. Each group consisted of 4 mice. For repeated doses, drugs were administered by IV injections every two or three days for a total of 10 injections. Body weight, deaths, changes in behaviour, motility, eating and drinking habits, and any other sign of local or systemic toxicity were recorded daily.

### Pharmacokinetics

Healthy CD-1 mice were injected IV with MTM-SK (18 mg/Kg) and MTM-SDK (2 mg/Kg). Blood was collected at five time-points between 5 and 120 min. At each time point 3 mice per group were analyzed. Plasma samples were diluted with 4 volumes of acetonitrile, vortexed and centrifuged to eliminate any insoluble precipitate. The supernatant was then used to measure MTM-SDK and MTM-SK by LC-MS. Plasma levels were assessed after intraperitoneal (IP) and IV injection of MTM-SK (18 mg/Kg) also by HPLC-UV.

### Gene expression analysis in tumor xenografts

To evaluate the effects on transcription in tumor tissues, mice bearing subcutaneous tumours were treated with MTM-SDK (1.2 mg/kg) and MTM-SK (8 mg/kg) or saline solution by IV injection. Animals were sacrificed after 1, 3 and 7 days from the injection. Tumors were immediately collected and snap frozen for RNA isolation. qRT-PCR was performed as described above. At each time point 4 mice were analyzed in each experimental group and qRT-PCR performed in triplicate for each sample.

### Antitumor activity

Mice bearing subcutaneous tumors were treated with compounds or vehicle. Each experimental group consisted of 10 mice. Drugs were prepared in sterile saline solution and given by IP injections every 3 days. Control mice received IP injection of sterile saline solution. Tumor size was measured with a caliper twice a week. Data represent average ± SD of 10 mice per group.

### Antimetastatic activity

To evaluate the compounds ability to block metastatic tumor growth, mice received tail vein injections of tumor cells to establish lung metastasis. Then mice were treated with MTM-SDK (1.2 mg/kg), MTM-SK (8 mg/kg) or sterile saline given by IP injections every 3 days starting two weeks after the tail vein injection of tumor cells. Two groups of untreated mice (*n = 3 per group*) were sacrificed after 1 and 2 weeks from the injection to confirm implantation and growth of metastatic cells. Control and drug treated mice (*n = 3 per group*) were then sacrificed after 1 and 2 weeks from the beginning of the treatment. Lung tissues were collected for qPCR analysis and histological examination after H&E staining. Lung metastases were quantified using a previously described qPCR based method [42]. Genomic DNA extracted from frozen lung tissues was amplified with primer sets for unique, conserved and species-specific regions of the human and mouse genome. PCR was carried out using SYBRGreen qPCR Master Mix [42]. The percentage of human cells in lung tissues was determined in triplicate paired reactions for each animal using standard curve of mixed human/mouse samples as described [42]. Data are mean ± SD of 3 mice per experimental point.

### Statistical analysis

All data are expressed as mean ± SD. The IC50 were calculated using SigmaPlot. Differences between experimental groups were analyzed for statistical significance using unpaired two-tailed T-test using GraphPad Prism 4.0 Software. P values≤0.05 were considered statistically significant.

## Results

### MTM-SDK and MTM-SK inhibit expression of Sp regulated genes in prostate cancer cells

**Figure 1:**
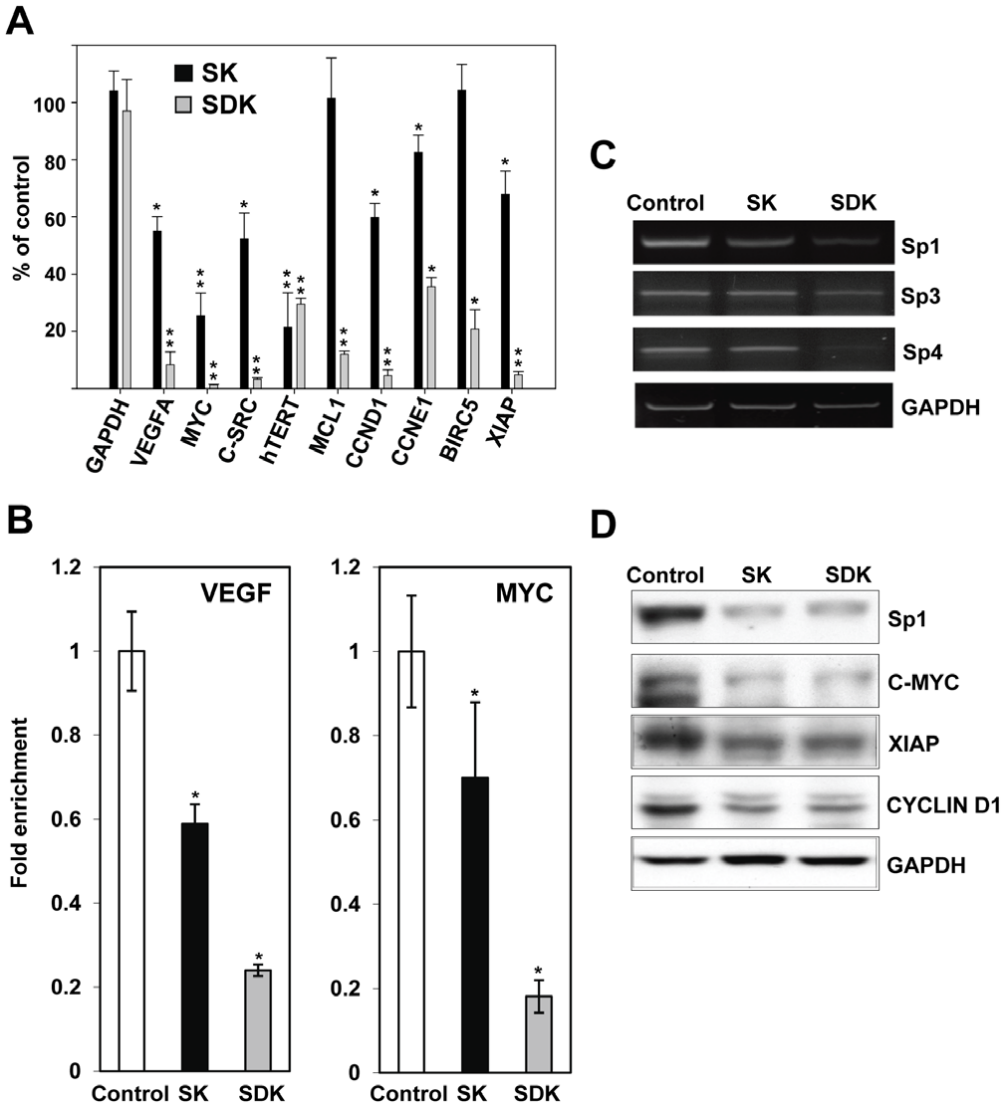
Inhibition of Sp regulated genes by MTM-SDK and MTM-SK in prostate cancer cells *in vitro*. PC3 cells were treated with 100 nM of MTM-SDK, MTM-SK or vehicle (DMSO) for 24 h. A) Gene expression was measured by qRT-PCR. Data were normalized to *B2M* RNA level and are presented as percentage of expression compared to vehicle-treated cells (control). Data represent the mean ± SD from 3 independent experiments. B) Binding of Sp1 to the promoters of *C-MYC* and *VEGF* in control and drug treated cells was determined by ChIP using an anti-Sp1 specific antibody. DNA in input and immunoprecipitated fractions was quantified by qPCR with primers encompassing the Sp binding site in the gene promoters. Data (mean ± SD) from 3 independent experiments are expressed as percentage of input DNA in immunoprecipated fractions. *, P<0.01; **, P<0.001. C) Level of Sp1, Sp3 and Sp4 mRNA was determined by RT-PCR in PC3 cells incubated with 100 nM of MTM-SDK, MTM-SK or vehicle for 24 and 48 h. GAPDH was used as control. D) Protein level of Sp1, c-Myc, XIAP, and cyclin D1 was determined by immunoblotting in PC3 cells incubated with 100 nM of MTM-SDK, MTM-SK or vehicle for 24 and 48 h.

Analysis of the current literature showed that many genes over-expressed in primary and metastatic prostate cancer are regulated by Sp TFs and are potential targets of the MTM-A analogues (see Methods S1, Table S1, S2 and references therein). Applying bioinformatics approaches to publicly available gene expression datasets we found that genes with predicted binding sites for Sp TFs in their promoters were frequently deregulated in prostate tumors both at the early and advanced stages of disease, despite the lack of direct evidence of over-expression of Sp TFs in these tumors (Fig. S1). Furthermore, we found that many genes down-regulated by MTM-SDK in an ovarian cancer cell line and putative targets of Sp TFs were over-represented among genes up-regulated in primary and metastatic prostate tumors (Fig. S1). Therefore, we assessed the impact of the new MTM-A analogues on transcription of Sp-regulated genes in prostate cancer cells. We selected genes known to be over-expressed in prostate tumors and involved in various aspects of prostate tumorigenesis like cell proliferation, apoptosis and angiogenesis (Table S1). Prostate cancer PC3 cells were treated with 100 nM of MTM-SDK, MTM-SK or vehicle for 24 h and the effects on gene expression were assessed by qRT-PCR. This dose was chosen on the basis of our previous work in ovarian cancer cells [27]. Furthermore, we verified that 24 h treatment at doses ≤100 nM was minimally cytotoxic (≤30% of reduction in cell viability; Fig. S2). Under these conditions, treatment with MTM-SDK reduced mRNA level of *C-MYC*, *hTERT*, *VEGFA*, *C-SRC*, *CCDN1*, *CCNE1*, *XIAP*, *MCL1* and *BIRC5* by ≥75% (Fig. 1A). As previously shown in ovarian cancer cells [27], MTM-SK was slightly less effective than MTM-SDK. The level of *C-MYC*, *hTERT*, *VEGFA*, *C-SRC* and *CCDN*1 mRNA was reduced in MTM-SK treated cells by 50–75% compared to control cells, while *XIAP*, *CCNE1*, *MCL1* and *BIRC5* were minimally or not affected.

MTM-A and its analogues bind to GC-rich DNA elements and prevent binding of GC-binding proteins like the Sp TFs to gene promoters. To determine whether the effects on transcription were due to inhibition of Sp binding , we measured the binding of Sp1 to the *C-MYC* and *VEGFA* promoter in control and drug treated cells by chromatin immunoprecipitation. MTM-SDK and MTM-SK significantly reduced binding of Sp1 to the *C-MYC* and *VEGFA* promoter (Fig. 1B). MTM-SDK was more effective than MTM-SK, in agreement with the gene expression data. As Sp proteins can control their own transcription [3], we evaluated whether MTM-SDK and MTM-SK affected their expression. Sp1, Sp3 and Sp4 are expressed in PC3 cells and treatment with the MTM-A analogues reduced the mRNA level of Sp1, Sp4 and to a lower extent Sp3 (Fig. 1C). Again, MTM-SDK was more effective MTM-SK. Protein levels of Sp1 and Sp targets, like c-Myc, were also reduced upon treatment with MTM-SDK- and MTM-SK in agreement with mRNA data (Fig. 1D).

### MTM-SDK and MTM-SK inhibit proliferation of prostate cancer cells

**Figure 2:**
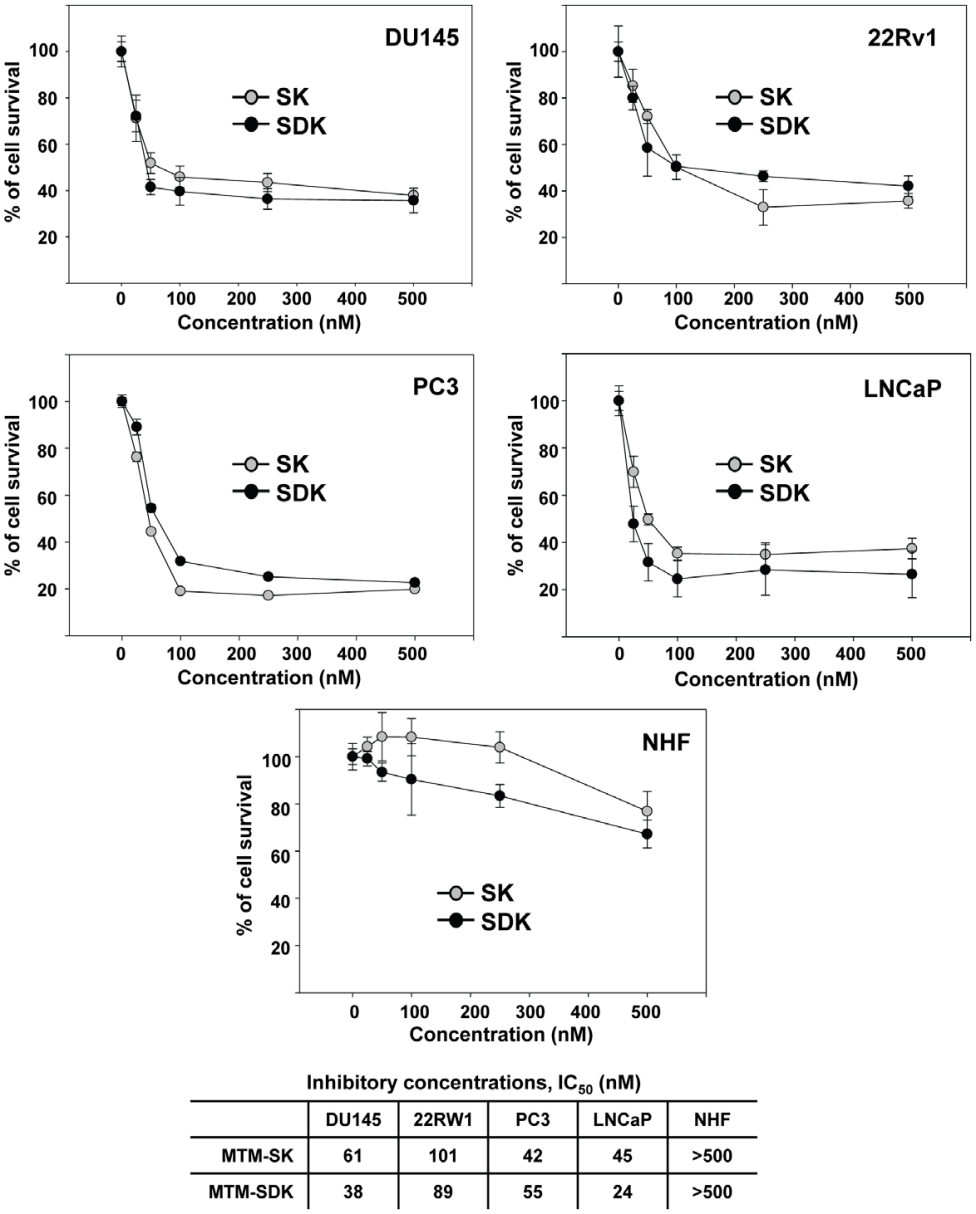
Antiproliferative effects of MTM-SDK and MTM-SK in prostate cancer cells *in vitro*. Prostate cancer cells (DU145, 22Rv1, PC3 and LNCaP) and primary cultures of normal human fibroblasts (NHF) were incubated with the compounds for 72 h. Cell viability was measured by the colorimetric MTT assay. Data are presented as mean ± SD of triplicate samples of 3 independent experiments. IC_50_ for each cell type are reported in the bottom panel.

Inhibition of Sp TFs has been shown to affect proliferation and survival of cancer cells. We examined the antiproliferative effects of MTM-SDK and MTM-SK in a panel of prostate cancer cell lines representing androgen-dependent (AD) and androgen-independent (AI) prostate tumors. LNCaP cells are androgen receptor (AR) positive and depend on AR signaling. 22Rv1 are AR positive but AI. PC3 and DU145 are AR negative and AI. Growth of all the cell lines tested, independent of their AR state, was strongly inhibited by both compounds, with MTM-SDK being more effective than MTM-SK (Fig. 2). The IC_50_ values are reported in Figure 2. Moreover, growth of prostate cancer cells was inhibited by ≥50% at ≤100 nM of both drugs. Notably, the drugs had no effect at these doses on normal human fibroblasts (Fig. 2). Viability of normal fibroblasts was affected slightly only at higher doses (∼20–30% of inhibition at 500 nM). This difference in sensitivity between normal and cancer cells was in line with our previous data showing that normal fibroblasts were more resistant than ovarian cancer cells to the induction of apoptosis when exposed to MTM-SDK [27].

### Toxicity of MTM-SDK and MTM-SK

Systemic toxicity is a major limitation to the clinical use of MTM-A. Therefore, we assessed the doses at which MTM-SDK and MTM-SK could be safely administered to mice upon both acute and chronic administration. The maximum tolerated doses (MTD) of MTM-SDK and MTM-SK after a single IV injection were 4 mg/kg and 32 mg/kg, respectively (Table 1). The MTD of MTM-SK for repeated treatment was 8 and 18 mg/kg using the q2d×10 and q3d×10 schedule, respectively. The MTD of MTM-SDK was 1.2 mg/Kg and 1.8 mg/Kg, respectively, when given with the q2d×10 and q3d×10 schedule. We tested for comparison MTM-A and found that the MTDs were 1 mg/kg and 0.5 mg/kg for single and repeated (q2d×10) administration, respectively. Further dose escalation of both MTM-SK than MTM-SDK and repeated treatment resulted in progressive loss of body weight and other signs of severe chronic toxicity, including decreased mobility, conjunctivitis and peripheral neuropathy. In conclusion, remarkably higher doses of MTM-SK than MTM-SDK compared to MTM-A could be given safely both as single and repeated injections to mice without signs of toxicity.

**Table 1:** Maximum tolerated doses of MTM compounds.

	Single dose (mg/kg)*	Repeated dose (mg/kg)*	
		q2d×10	q3d×10
MTM-SK	32	8	18
MTM-SDK	4	1.2	1.8
MTM	1	0.5	ND

* CD-1 mice received IV single injections of MTM-SK, MTM-SDK and MTM or repeated injections of the compounds every two (q2d×10) or three days (q3d×10) for a total of 10 injections. *ND*, not determined.

### Pharmacokinetics of MTM-SDK and MTM-SK

**Figure 3:**
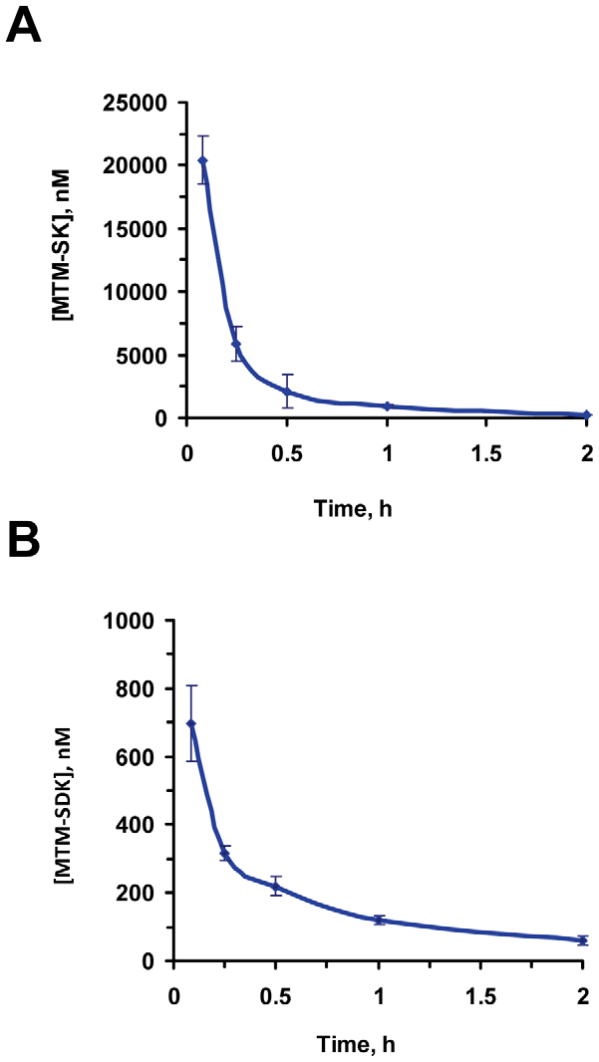
Pharmacokinetics profile of MTM-SDK and MTM-SK in mice. Mice (n = 3/group) received a single IV injection of MTM-SK (A) or MTM-SDK (B) and plasma levels were determined by LC-MS. Doses of MTM-SK and MTM-SDK were 18 mg/kg and 2 mg/kg, respectively.

Blood levels of MTM-SK and MTM-SDK were measured after single IV injections at the dose of 18 and 2 mg/kg (Fig. 3). Both compounds were cleared rapidly from the bloodstream with similar overall kinetics. After 5 min plasma levels were about 20 and 0.7 µM for MTM-SK and MTM-SDK, respectively. After 1 h the levels of MTM-SK and MTM-SDK were approximately 1 and 0.1 µM, respectively. Thus, plasma levels of both compounds were at or above the concentration required *in vitro* for cell growth inhibition and suppression of Sp1 dependent transcription. Next, we compared pharmacokinetics of MTM-SK following single IV and IP injections. Beside the initial peak seen with the IV injection, the kinetics following IV and IP administration were quite similar, reaching comparable drug levels in the plasma within 15–30 min and maintaining similar levels over the 2-h period of the analysis (Fig. S3).

### MTM-SDK and MTM-SK inhibit gene expression in human prostate tumor xenografts

**Figure 4:**
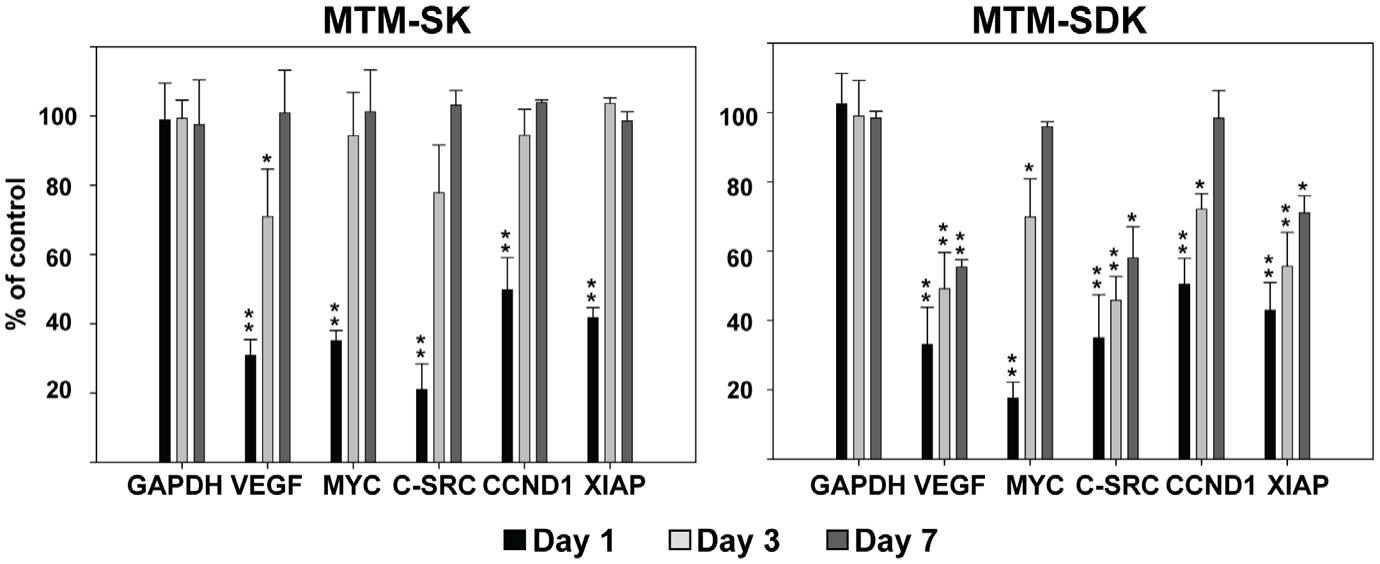
Inhibition of Sp dependent transcription by MTM-SDK and MTM-SK in tumor xenografts. Mice (n = 4/group) with subcutaneous tumors were treated with a single IV injection of MTM-SDK, MTM-SK or sterile saline solution. Doses of MTM-SK and MTM-SDK were 8 mg/kg and 1.2 mg/kg, respectively. Tumors were harvested 1, 3 and 7 days after drug injection. Gene expression was assessed by qRT-PCR. Data represent mean ± SD of the transcript level normalized to B2M RNA and are presented as percentage of expression compared to control mice receiving sterile saline solution. *, P<0.01; **, P<0.001.

To determine whether the MTM-A analogues were able to interfere with the activity of Sp TFs in vivo upon systemic administration, mice bearing PC3 tumor xenografts were treated with a single IV injection of MTM-SDK (1.2 mg/kg), MTM-SK (8 mg/kg) or saline solution. Tumors were excised at 1, 3 and 7 days after the injection and RNA levels of selected Sp regulated genes were measured by qRT-PCR. Expression of *C-MYC*, *hTERT*, *VEGFA*, *C-SRC*, *CCDN1*, *CCNE1*, *XIAP*, *MCL1* and *BIRC5* was significantly decreased by both MTM-SDK and MTM-SK at 24 h (Fig. 4). However, there was a difference among the two compounds in the kinetics of recovery from transcriptional inhibition. The effect of MTM-SDK lasted longer. Most genes were still repressed by 30 to 50% after 3 days. Even after 7 days *VEGFA*, *C-SRC* and *XIAP* were still significantly down-regulated. The effect of MTM-SK was reversed more rapidly with most genes returning to control levels within 3 or 7 days. Thus, both drugs were effective in reducing transcription of Sp-regulated genes when given systemically to mice, confirming the accumulation of active drug concentrations in the tumor tissue. Furthermore, the effect of MTM-SDK was more pronounced and persistent compared to MTM-SK, consistent with *in vitro* data on Sp1 binding and gene expression.

### MTM-SDK and MTM-SK inhibit growth of prostate tumor xenografts

**Figure 5:**
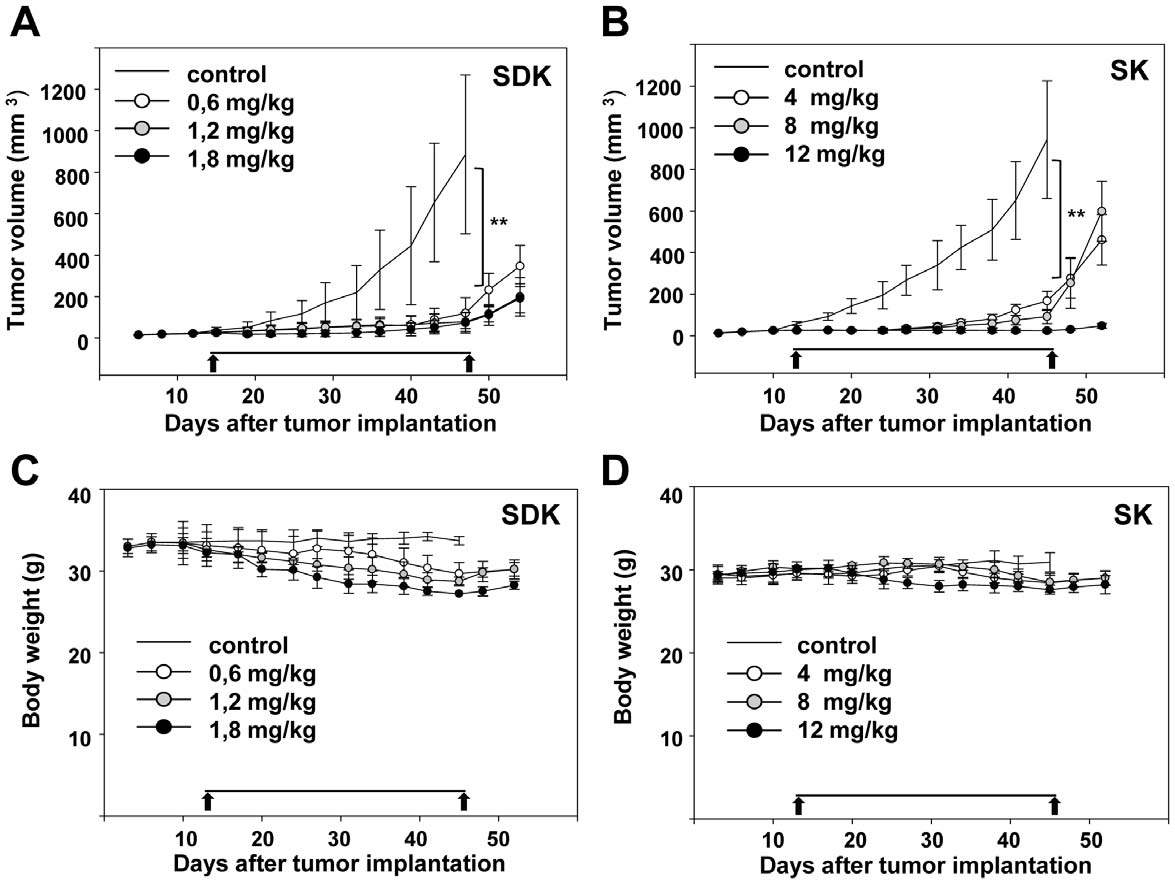
Antitumor activity of MTM-SDK and MTM-SK in subcutaneous prostate tumor xenografts. Mice with subcutaneous tumors were treated with the indicated doses of MTM-SDK, MTM-SK or sterile saline solution (control) given by IP injections twice a week for five weeks (q3d×10). Tumor volume (A) and body weight (B) were measured twice a week. Results are expressed as mean ± SD of the tumor volume in each group. Arrows indicate start and end of the treatment. **, P<0.001.

The anti-tumor activity of MTM-SDK and MTM-SK was examined using subcutaneous tumor xenografts of PC3 prostate cancer cells. PC3 cells are AI, highly tumorigenic and metastatic. Thus, these cells represent a good model of advanced castration-resistant prostate cancer. Mice bearing PC3 tumor xenografts received MTM-SDK (0.6, 1.2 and 1.8 mg/kg, q3d×10) and MTM-SK (4, 8 and 12 mg/kg,) every 3 days by IP injections. The IP administration was chosen as it is commonly used for prolonged treatments with anticancer compounds. Furthermore, IV and IP injections were nearly equivalent in terms of plasma levels and pharmacokinetics. Doses and schedule of administration were also based on the pharmacodynamic and MTD data described above. The treatment was terminated when mice in control groups had to be sacrificed due to excessive tumor burden. Drug-treated mice were followed for another week after the end of the treatment. Treatment with MTM-SDK and MTM-SK reduced tumor growth (Fig. 5A–B). The difference between treated and untreated mice was highly statistically significant at all doses tested. After the treatment was discontinued, growth of the subcutaneous tumors slowly resumed with a slightly faster kinetics in the case of the lower doses of MTM-SK. This is in agreement with pharmacodynamic data, indicating a faster recovery of gene expression after MTM-SK treatment. Interestingly, however, minimal tumor regrowth was seen in mice treated with the highest dose of MTM-SK. Treatment with MTM-SDK and MTM-SK resulted in slight loss of body weight, which was correlated with the dose level but not statistically significant (Fig. 5C–D). No signs of acute toxicity or local irritation at the site of injection were observed during treatment. Two mice treated with the highest dose of MTM-SDK (1.8 mg/kg) showed signs of toxicity (e.g., decreased mobility, peripheral neuropathy) after multiple injections. These mice were taken off treatment and were not included in the assessment of antitumor activity. Since the same dose was given safely to non-tumor bearing mice, the toxicity observed at this dose could be due to the presence of the tumor or differences in mice strain or sex.

### MTM-SDK and MTM-SK inhibit growth of metastatic prostate cancer

**Figure 6:**
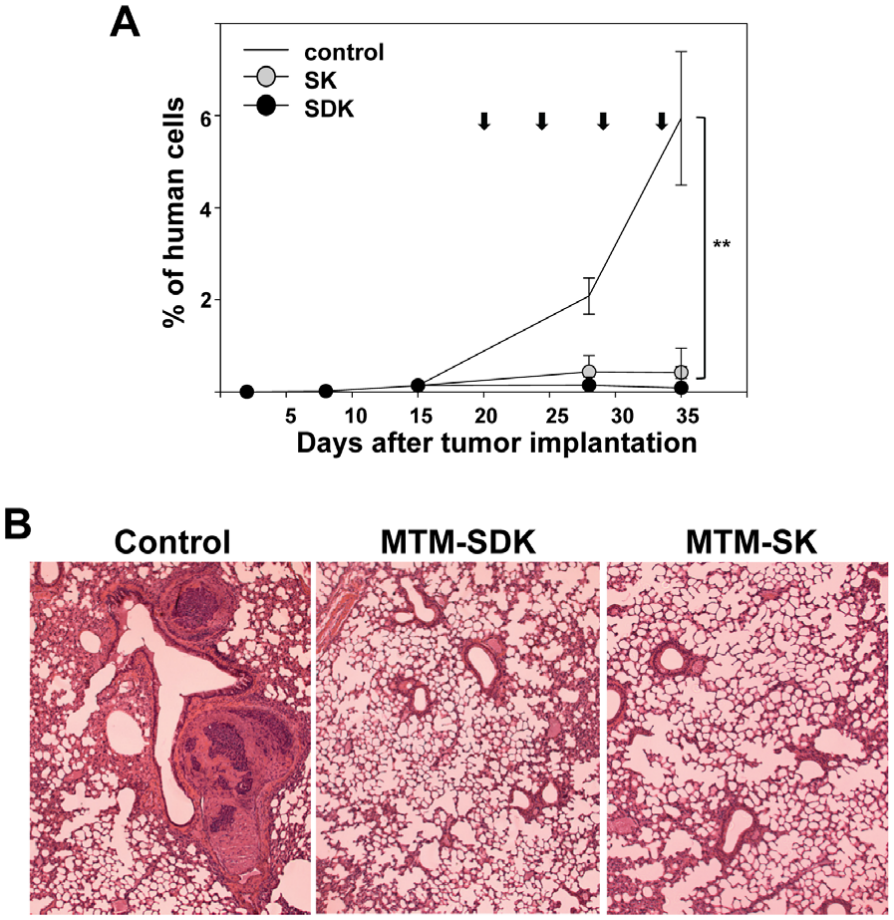
Inhibition of prostate cancer metastasis to the lung by MTM-SDK and MTM- SK. Mice were given PC3 by IV injection into tail vein and then starting 3 weeks later received IP injections of MTM-SDK, MTM-SK or sterile saline solution twice a week for two weeks. The doses of MTM-SK and MTM-SDK were 8 mg/kg and 1.2 mg/kg, respectively. A) qPCR assessment of lung metastasis. Control mice were sacrificed 1 and 2 weeks before treatment and thereafter control and drug treated mice were sacrificed at end of the first and second week of treatment. Results are presented as mean ± SD of the percentage of human cells relative to total mouse cells in the lung (n = 3). Arrows indicate the time of drug injections. B) Microscopic evaluation of lung metastasis. Lung tissue was collected from control and drug-treated mice at the end of the experiment and stained with H&E for histological examination. Representative sections are shown (×20). **, P<0.001.

We were interested in determining whether treatment with the MTM-A analogues was also effective in a metastatic model of prostate cancer. To this end, we used an experimental lung metastasis model to study the effects of the treatment on disseminated human cancer cells in immunodeficient mice. PC3 cells were injected in the tail vein of mice and the growth of lung metastasis was monitored by qPCR over a 4-week period [42]. A group of untreated control mice was sacrificed after the first and second week from the injection of tumor cells. At this time, the lungs contained 0.015 and 0.135% of human cells, respectively, confirming the implantation and proliferation of the injected human cancer cells (Fig. 6A). Thereafter, mice received every 3 days IP injections of MTM-SDK (1.2 mg/kg), MTM-SK (8 mg/kg) or vehicle . Groups of control and drug-treated mice were sacrificed two days after the second and fourth injection and their lungs examined for the presence of metastasis. At this time, control mice contained 2.08% and 5.94%, respectively, of human cancer cells consistent with the expansion of the metastatic foci in the lung (Fig. 6A). Instead, mice treated with MTM-SDK had 0.14% and 0.09% and those treated with MTM-SK had 0.43% and 0.42% of human cancer cells after the second and fourth injection, respectively. Thus, the growth of disseminated human metastatic cells was almost completely suppressed by the treatment. The qPCR data were confirmed by histological examination of lung sections taken at the end of the experiment (Fig. 6B). Multiple metastatic nodules were detected in the lung of control mice, while there were no metastases detectable by microscopic examination of serial sections of lungs of the drug treated animals.

## Discussion

Sp family transcription factors control many cellular processes essential for development of human tumors [3]. Diverse strategies have been explored in recent years to interfere with the activity of Sp TFs including natural compounds and small interfering RNAs. Aureolic acid derivatives, like MTM-A, are effective inhibitors of Sp TFs [15], [18], [19], [20]. These compounds bind to GC-rich DNA and prevent the interaction of the TFs with GC-rich sequences in gene promoters. In addition, since Sp TFs control their own transcription, MTM-A reduces the level of Sp proteins, thus reinforcing the effect on transcriptional activity [21], [34]. Various factors can influence the pharmacological and toxicological properties of the aureolic antibiotics: affinity for GC-rich sequences, association/dissociation kinetics to DNA, ability to compete with Sp proteins for binding to DNA and cellular uptake [17], [20], [27], [43]. These biochemical and biophysical properties ultimately determine the efficacy of this class of compounds as Sp TF inhibitors and their specificity toward Sp regulated genes and selectivity toward cancer cells [27]. Differences in metabolism and tissue distribution could also contribute to distinct *in vivo* pharmacological and toxicological profiles. Genetic manipulation of the MTM-A biosynthetic pathway to generate new compounds with an array of structural modifications may provide the means to produce analogues more active and more suitable for clinical use than MTM-A [23], [24], [25]. In the present study we investigated two MTM-A analogues, named MTM-SDK and MTM-SK, obtained using this metabolic engineering approach [27]. Recently we showed that MTM-SDK and MTM-SK exhibited increased potency as inhibitors of Sp TFs and as anticancer agents compared to MTM-A in biochemical and cellular assays [27]. Furthermore, MTM-SDK and MTM-SK exhibited relevant in vivo antitumor activity in human ovarian tumor xenografts [29].

In this study, we investigated the pharmacological activity of MTM-SDK and MTM-SK in prostate cancer cell lines and prostate tumor xenografts. The two compounds were effective inhibitors of the transcription of Sp1-regulated genes both in vitro and in vivo. Toxicity studies revealed that MTM-SDK and MTM-SK were well tolerated upon systemic administration in mice. The MTDs for single injections of MTM-SDK and MTM-SK were, respectively, 4- and 32- fold higher than that of MTM-A. Repeated treatment with both compounds using two different schedules of administration was also well tolerated. Using doses and schedules of treatment that could be safely administered , both compounds exhibited potent antitumor activity in subcutaneous prostate tumor xenografts. Even at the lowest dose tested, treatment with MTM-SDK and MTM-SK significantly reduced tumor growth. Prolonged treatment was well tolerated also in tumor bearing mice with minimal reduction of body weight. Only at the highest dose of MTM-SDK there were signs of toxicity in few animals at the end of the treatment. MTM-SDK and MTM-SK were also very active in a metastatic prostate cancer xenograft model. Although lung is not a primary site for prostate cancer, this experimental model approximates the clinical situation of advanced disease with multiple metastatic foci growing at ectopic sites. Advanced prostate cancer is difficult to treat and the finding that the MTM analogues arrested the expansion of metastatic foci in the lung could be clinically relevant. The metastatic cascade comprises several steps. This experimental model requires cancer cells to survive in the bloodstream, extravasate, form micro-metastasis and then expand into clinically evident macro-metastasis. In our experiments treatment was initiated at the stage when multiple micro-metastatic foci were present in the lung of control animals [42]. MTM-SDK and MTM-SK were able to block further expansion of the metastatic foci, leading to almost complete arrest of their growth. The mechanisms underlying the antimetastatic activity of the MTM-A analogues would need to be investigated further. Various metastasis promoting genes involved in multiple steps of the metastatic cascade are known to be regulated by Sp1 [44]. Thus, it is likely that the effect depends on the ability of the compounds to block expression of genes necessary for implantation, survival and expansion of the metastatic clones.

We assessed pharmacokinetics and pharmacodynamics properties of the MTM-A analogues to guide the choice of dose and schedule of administration for the antitumor activity assays. We found that MTM-SK and MTM-SDK were cleared rapidly from the bloodstream after IV administration according to a typical exponential decay. The pharmacodynamic data suggest a rapid uptake in cells and tissues that would compensate for the short half-life in the bloodstream as indicated also by previous *in vitro* studies [27]. With both compounds there was no evidence of metabolites in the blood, indicating that the decay was due to clearance rather than metabolism. IP injection, which was used for antitumor activity assay, was equivalent to IV injection in terms of plasma levels. In the case of MTM-SK, initial plasma concentrations after IP administration were low, increased to reach a maximum within 15–30 min and then rapidly decreased mimicking the behavior seen after IV injection. The pharmacodynamic analysis of the effect on Sp dependent transcription showed that both compounds inhibited transcription at 24 h after injection. Interestingly, there was a difference among the two compounds in the recovery from the transcriptional inhibition. Most genes were equally repressed at day 1 and day 3 in MTM-SDK treated mice. Even at day 7 many genes were still significantly repressed. On the other hand, the effect of MTM-SK on transcription was lost more rapidly. This is consistent with the differences in DNA binding and inhibition of Sp1 activity seen previously between the two compounds in cell culture experiments [27]. Differences in affinity and reversibility of the binding to DNA among the aureolic acid derivatives could play an important part in determining the differences in biological activity [17], [27], [43]. Other properties might also be at play: cellular uptake and nuclear retention are also different between MTM-SDK and MTM-SK [27]. It is interesting that tumor growth also recovered more rapidly in MTM-SK than MTM-SDK treated mice when treatment was ended, again suggesting more rapid recovery from the effects of MTM-SK. Thus, distinct properties of the two compounds could impact on their ability to sustain inhibition of Sp dependent transcription and tumor growth and could modulate as well their potential toxicity. The faster recovery of the effects of MTM-SK, which could be attributed to a less tight and more readily reversible binding to DNA, might in fact be advantageous in terms of limiting the drug's potential toxicity. Indeed, there was a significant difference between the doses of MTM-SDK and MTM-SK that could be safely given to mice, although both compounds were more tolerated and could be administered at higher doses than the parent compound.

The activity of MTM-SDK and MTM-SK demonstrated here in prostate cancer cell lines and prostate tumor xenografts is highly encouraging. Along with previous data in other tumor types, these results indicate that these MTM-A analogues could be good candidates for treatment of cancers in which Sp TFs play an important role in driving the disease. Recent studies also have expanded the potential of Sp TF targeting compounds like MTM-A as cancer therapeutics. MTM-A was shown to have synergistic activity in combination with other anticancer drugs in various experimental models of human cancers [21], [34], [45]. Furthermore, genetic alterations (e.g., single nucleotide polymorphisms in gene promoters [46], [47], [48] and chromosomal translocations leading to the production of aberrant TFs [49]) that increase the risk of cancer initiation and progression could determine increased sensitivity of cancer cells to MTM-A. The availability of safer MTM-A analogues, like those described here, could lead to translation of these laboratory observations into clinical applications. In the case of prostate cancer, although there is no evidence of over-expression of Sp TFs, numerous lines of evidence suggest that Sp1 and other Sp TFs are important players. We found that several Sp regulated genes are over-expressed in prostate tumors (Table S1) and compounds that interfere with the activity of Sp TFs have antitumor or chemo-preventive activity in this disease. Furthermore, a bioinformatics analysis of gene profiling data from prostate tumor samples at different stages of disease indicated the involvement of Sp TFs in the transcriptional perturbations associated with primary and metastatic disease (see Methods S1 and Fig. S1). Comparing the up-regulated genes in primary and metastatic prostate cancers with the MTM-SDK down-regulated genes identified in a previous gene profiling study [27], we found a significant overlap suggesting that many genes deregulated in prostate cancer are potential targets of this class of compounds (Fig. S1). A similar approach could be used to identify other tumor types potentially sensitive to Sp TF targeting strategies. Collectively, these data provide further evidence of the relevance of Sp TFs as therapeutic targets and of the potential of MTM-A analogues as effective cancer therapeutics.

## Supporting Information

Methods S1 Bioinformatics analysis of Sp transcription factors in prostate cancer.
(DOC)

Figure S1 Gene set enrichment analysis of Sp target genes in prostate cancer.
(PDF)

Figure S2 Cell viability following 24-h incubation with MTM-SK and MTM-SDK.
(PDF)

Figure S3 Pharmacokinetics profile of MTM-SK following intravenous (IV) and intraperitoneal (IP) injection in mice.
(PDF)

Table S1 Genes with experimental evidence of control by Sp transcription factors and implication in prostate cancer development according to analysis of recent literature.
(PDF)

Table S2 Microarrays data sets downloaded from GEO.
(PDF)

Table S3 PCR primer sets and sequences.
(PDF)
