# Supplementary material for: Modulation of the Activity of Sp Transcription Factors by Mithramycin Analogues as a New Strategy for Treatment of Metastatic Prostate Cancer
Source: PLoS One. 2012 Apr 19;7(4):e35130. doi: 10.1371/journal.pone.0035130 (PMC3334962; doi:10.1371/journal.pone.0035130)
Supplement: Methods S1 — Bioinformatics analysis of Sp transcription factors in prostate cancer. (DOC) [file pone.0035130.s001.doc]

**Supporting information**

**Supplementary methods and data: Bioinformatics analysis of Sp transcription factors in prostate cancer**

***Microarray data retrieval and bioinformatics analysis.*** Microarray data from various gene profiling studies (see Table S2) were downloaded from GEO (<http://www.ncbi.nlm.nih.gov/geo/>). The downloaded data were MAS 5.0 normalized in the R statistical environment ([http://www.R-project.org](http://www.R-project.org/)) using the Affy Bioconductor package ([http://www.bioconductor.org](http://www.bioconductor.org/)). The data were then imported into BRB-ArrayTools 3.8.0_beta1 (<http://linus.nci.nih.gov/BRB-ArrayTools.html>) developed by Simon and colleagues (1). Individual spots were excluded from the analysis if the intensity was ≤10. Genes were excluded if ≥1.5-fold change from the median was seen in ≤ 20% of samples or the data missing or filtered out were ≥ 50%. Data were normalized using the median over the entire arrays. Group comparison analysis was used to determine expression differences between normal prostate and prostate cancer, between primary and metastatic prostate cancer, and between androgen-dependent and androgen-independent prostate cancer.

Gene set enrichment analysis (GSEA; http://www.broad.mit.edu/gsea) was performed using the software developed by Subramanian and colleagues (2). GSEA is a supervised method to assess the differential expression (enrichment) of pre-defined gene sets in gene expression datasets. Gene sets used in the analysis were: a) genes with predicted Sp1 binding sites in their promoters (V$SP1_Q2_01; GGGGCGGGGN) and b) genes down-regulated by MTM-SDK in ovarian cancer cells [1]. The gene set (V$SP1_Q2_01) was downloaded from Molecular Signatures Database (MSigBD) consisting of transcription factor targets gene sets file ([c5.mf.v2.5.symbols.gmt](http://www.broadinstitute.org/gsea/msigdb/download_file.jsp?filePath=/msigdb/downloads/c5.mf.v2.5.symbols.gmt)). The other gene set (MTM-SDK down-regulated) was created and added to the same gene sets file. GSEA ranks the genes in the dataset based on their correlation with the two phenotypes being compared. Then, it identifies the rank positions of the members of the specific gene set in relation to the phenotype and calculates an enrichment score (ES), which reflects the degree to which a gene set is over-represented at the extremes. A thousand permutations were used to test the significance of enrichment and calculate the p-values.

***Sp regulated genes and potential targets of Sp inhibitors in prostate cancer.*** A systematic analysis of the current literature revealed that many genes implicated in prostate tumorigenesis are potential targets of the Sp transcription factors (TF). To confirm this finding, we used a bioinformatics approach to evaluate the expression of putative Sp regulated genes and potential targets of Sp TF inhibitors in prostate tumors at various stages of progression.

To identify genes associated with prostate cancer initiation and progression we compared publicly available microarray datasets from different studies (see Table 1) that included samples of normal prostate tissue (*n=18*) and primary (*n=65*), metastatic (*n=24*), androgen-dependent (*n=10*) and androgen-independent (*n=10*) tumors. Comparing all the different microarray datasets we did not find statistically significant changes in the expression of Sp TFs. However, since functional activation of a transcription factor can be reflected in the activation of its target genes, we examined the distribution of the putative Sp target genes among the genes implicated in prostate cancer development and progression using Gene Set Enrichment Analysis. The gene sets that were used in the analysis were a) genes having predicted Sp binding sites in their promoter, and b) genes down-regulated by MTM-SDK as defined in a previous study in ovarian cancer cells (3). Genes that were differentially expressed in prostate tumors relative to normal prostate were enriched of genes with predicted Sp binding sites in the promoter (Figure S1A). Interestingly, both up- and down-regulated genes showed a significant association with the presence of putative Sp1 binding sites (ES=0.49; P=0.001). Thus, Sp TF functions during prostate cancer development could be bi-directional and equally relevant for both transcriptional activation and repression. We examined also genes differentially expressed during progression to metastatic and androgen-independent disease. Progression of prostate cancer from primary to metastatic tumors was also associated with transcriptional activation of potential Sp targets, as we found enrichment of genes with predicted Sp1 binding sites among up-regulated genes in metastatic tumors (ES=0.36; P=0.057). The same tendency, however not statistically significant, was observed when comparing androgen-dependent vs. androgen-independent prostate cancers. Together, these findings indicated that Sp target genes are frequently over-expressed in both primary and metastatic prostate tumors reflecting over-activity of Sp TFs. Thus, these genes may represent potential targets of Sp TF inhibitors. Therefore, we sought to determine whether genes affected by MTM analogues like MTM-SDK were enriched among the up-regulated genes in prostate tumors. Indeed, comparison with this list of the potential targets of Sp TF inhibitors revealed that the genes down-regulated by MTM-SDK were significantly over-represented (ES=0.44; P=0.01) among the genes up-regulated in primary prostate tumors (Figure S1B). Thus, Sp TF inhibitors like MTM-SDK could be able to repress transcription of Sp regulated genes activated in prostate tumors.

**References**

1. Simon R, Lam A, Li MC, Ngan M, Menenzes S, Zhao Y. Analysis of Gene Expression Data Using BRB-Array Tools. Cancer informatics 2007; 3:11-7.

2. Subramanian A, Tamayo P, Mootha VK, *et al.* Gene set enrichment analysis: a knowledge-based approach for interpreting genome-wide expression profiles. Proc Natl Acad Sci U S A, 2005. **102**(43): p. 15545-50.

3. Albertini V, Jain A, Vignati S*, et al.* Novel GC-rich DNA-binding compound produced by a genetically engineered mutant of the mithramycin producer Streptomyces argillaceus exhibits improved transcriptional repressor activity: implications for cancer therapy. Nucleic Acids Res 2006; 34(6):1721-34.

4. Yu YP, Landsittel D, Jing L*, et al.* Gene expression alterations in prostate cancer predicting tumor aggression and preceding development of malignancy. J Clin Oncol 2004; 22(14):2790-9.

5. Chandran UR, Ma C, Dhir R*, et al.* Gene expression profiles of prostate cancer reveal involvement of multiple molecular pathways in the metastatic process. BMC cancer 2007; 7:64.

6. Best CJ, Gillespie JW, Yi Y*, et al.* Molecular alterations in primary prostate cancer after androgen ablation therapy. Clin Cancer Res 2005; 11:6823-34.
