# Supplementary material for: Modulation of the Activity of Sp Transcription Factors by Mithramycin Analogues as a New Strategy for Treatment of Metastatic Prostate Cancer
Source: PLoS One. 2012 Apr 19;7(4):e35130. doi: 10.1371/journal.pone.0035130 (PMC3334962; doi:10.1371/journal.pone.0035130)
Supplement: Figure S1 — Gene set enrichment analysis of Sp target genes in prostate cancer. (PDF) [file pone.0035130.s002.pdf]

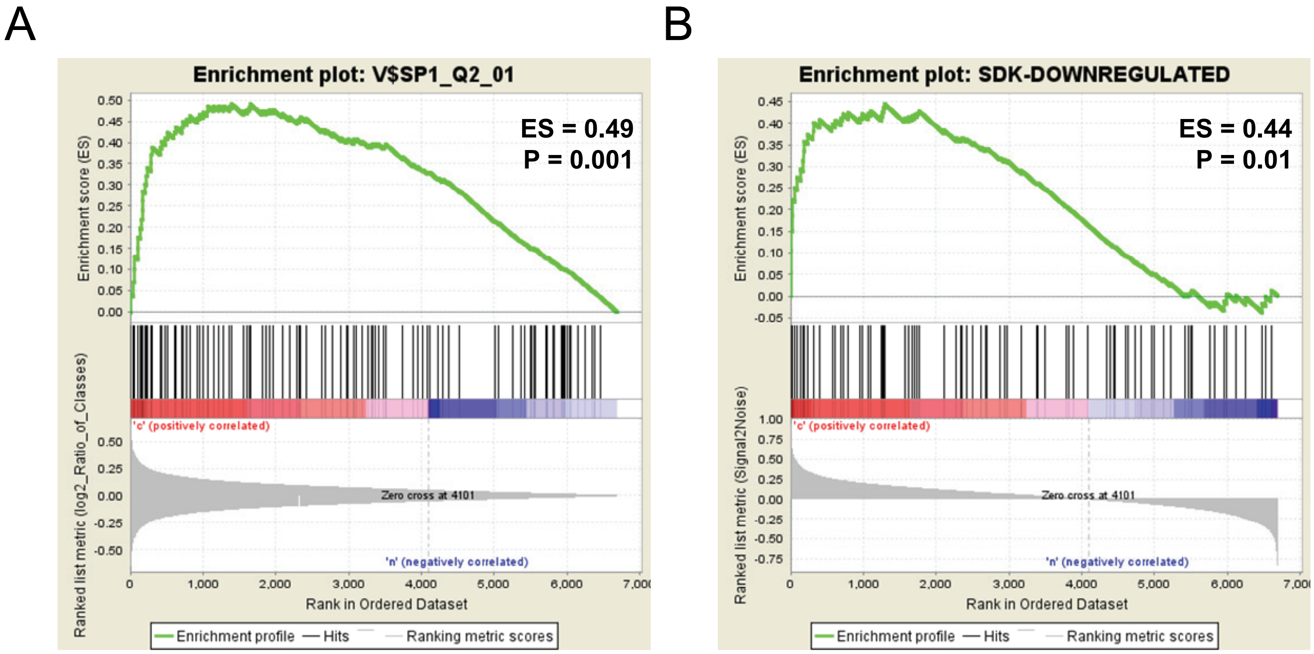

Figure S1. Gene set enrichment analysis of Sp target genes in prostate cancer. Correlation of tested gene sets was assessed by maximum enrichment score (ES) and the significance was assessed using permutation testing. (A) Enrichment of genes with predicted Sp1 binding site in the promoter region among genes differentially expressed in prostate cancer vs. normal prostate (“c” vs. “n”). (B) Enrichment of genes down-regulated by MTM-SDK among genes over-expressed in prostate cancer compared to normal prostate (“c” vs. “n”).
