# Supplementary material for: Modulation of the Activity of Sp Transcription Factors by Mithramycin Analogues as a New Strategy for Treatment of Metastatic Prostate Cancer
Source: PLoS One. 2012 Apr 19;7(4):e35130. doi: 10.1371/journal.pone.0035130 (PMC3334962; doi:10.1371/journal.pone.0035130)
Supplement: Figure S2 — Cell viability following 24-h incubation with MTM-SK and MTM-SDK. (PDF) [file pone.0035130.s003.pdf]

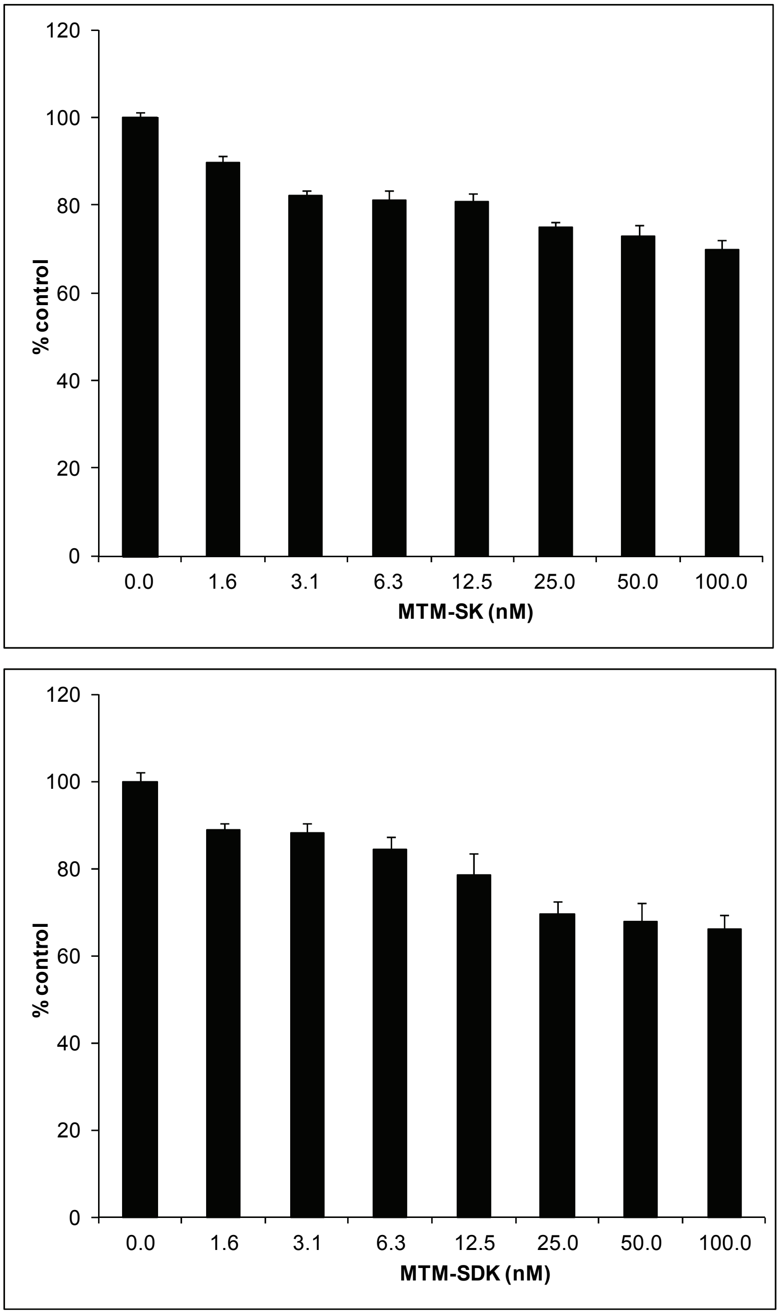

Figure S2. Cell viability following 24-h incubation with MTM-SK and MTM-SDK. PC3 cells were incubated with the indicated dose of the compounds or vehicle (DMSO). Cell viability was measured by the colorimetric MTT assay after 24 h. Data represent mean ± SD of 6 replicates from two independent experiments.
