# Supplementary material for: Modulation of the Activity of Sp Transcription Factors by Mithramycin Analogues as a New Strategy for Treatment of Metastatic Prostate Cancer
Source: PLoS One. 2012 Apr 19;7(4):e35130. doi: 10.1371/journal.pone.0035130 (PMC3334962; doi:10.1371/journal.pone.0035130)
Supplement: Figure S3 — Pharmacokinetics profile of MTM-SK following intravenous (IV) and intraperitoneal (IP) injection in mice. (PDF) [file pone.0035130.s004.pdf]

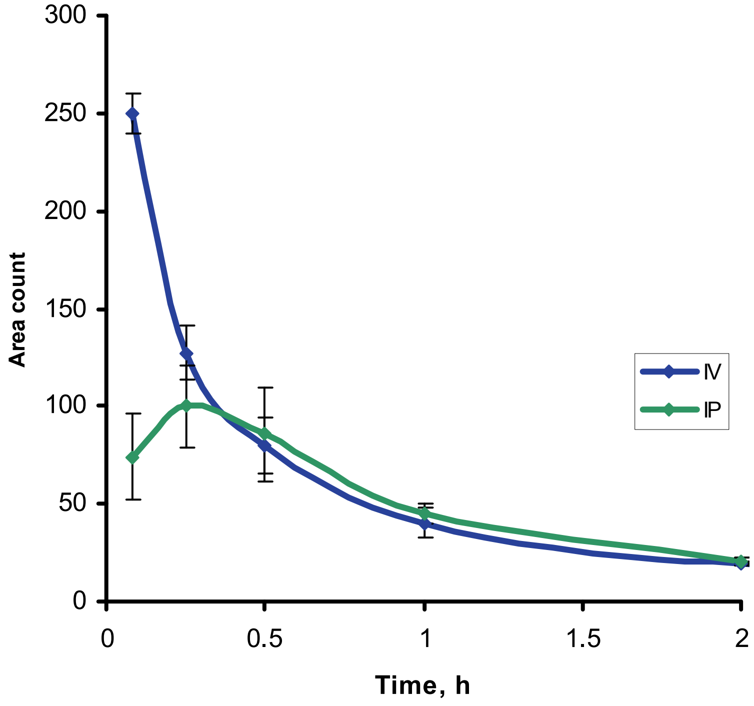

Figure S3. Pharmacokinetics profile of MTM-SK following intravenous (IV) and intraperitoneal (IP) injection in mice. Mice (n=3/group) received a single injection of 18 mg/Kg of MTM-SK and plasma levels were determined by HPLC-UV.
