# Supplementary material for: Modulation of the Activity of Sp Transcription Factors by Mithramycin Analogues as a New Strategy for Treatment of Metastatic Prostate Cancer
Source: PLoS One. 2012 Apr 19;7(4):e35130. doi: 10.1371/journal.pone.0035130 (PMC3334962; doi:10.1371/journal.pone.0035130)
Supplement: Table S1 — Genes with experimental evidence of control by Sp transcription factors and implication in prostate cancer development according to analysis of recent literature. (PDF) [file pone.0035130.s005.pdf]

Table S1. Genes with experimental evidence of control by Sp transcription factors and implication in prostate cancer development according to analysis of recent literature

Gene simbol	Gene ID	References	
		Control by Sp1	Implication in PCa
VEGFA	7422	(1, 2)	(3)
MYC	4609	(4)	(5, 6)
C-SRC	6714	(7)	(6, 8)
hTERT	7015	(9)	(10)
MCL1	4170	(11)	(12)
CCND1	595	(13)	(14)
CCNE1	898	(15)	(16)
CLDN4	1364	(17)	(18)
BIRC5	332	(19)	(20)
XIAP	331	(21)	(22)
MMP14	4323	(23)	(23)
C-FOS	2353	(24)	(25)
AR	367	(26, 27)	(27)
KLK3	354	(28)	(28)

References

1. Shi Q, Le X, Abbruzzese JL, et al. Constitutive Sp1 activity is essential for differential constitutive expression of vascular endothelial growth factor in human pancreatic adenocarcinoma. Cancer Res 2001;61:4143-54.
2. Abdelrahim M, Smith R, 3rd, Burghardt R, Safe S. Role of Sp proteins in regulation of vascular endothelial growth factor expression and proliferation of pancreatic cancer cells. Cancer Res 2004;64:6740-9.
3. Yao JL, Ryan CK, Francis CW, Kohli M, Taubman MB, Khorana AA. Tissue factor and VEGF expression in prostate carcinoma: a tissue microarray study. Cancer Invest 2009;27:430-4.
4. Parisi F, Wirapati P, Naef F. Identifying synergistic regulation involving c-Myc and sp1 in human tissues. Nucleic Acids Res 2007;35:1098-107.
5. Nagy B, Szendroi A, Romics I. Overexpression of CD24, c-myc and Phospholipase 2A in Prostate Cancer Tissue Samples Obtained by Needle Biopsy. Pathol Oncol Res 2009;15:279-83.
6. Creighton CJ. Multiple oncogenic pathway signatures show coordinate expression patterns in human prostate tumors. PLoS ONE 2008;3:e1816.

7. Ritchie S, Boyd FM, Wong J, Bonham K. Transcription of the human c-Src promoter is dependent on Sp1, a novel pyrimidine binding factor SPy, and can be inhibited by triplex-forming oligonucleotides. J Biol Chem 2000;275:847-54.
8. Lara PN, Jr., Longmate J, Evans CP, et al. A phase II trial of the Src-kinase inhibitor AZD0530 in patients with advanced castration-resistant prostate cancer: a California Cancer Consortium study. Anticancer Drugs 2009;20:179-84.
9. Bermudez Y, Yang H, Saunders BO, Cheng JQ, Nicosia SV, Kruk PA. VEGF- and LPA-induced telomerase in human ovarian cancer cells is Sp1-dependent. Gynecol Oncol 2007;106:526-37.
10. Tang J, Wang Z, Li X, Li J, Shi H. Human telomerase reverse transcriptase expression correlates with vascular endothelial growth factor-promoted tumor cell proliferation in prostate cancer. Artif Cells Blood Substit Immobil Biotechnol 2008;36:83-93.
11. Townsend KJ, Zhou P, Qian L, et al. Regulation of MCL1 through a serum response factor/Elk-1-mediated mechanism links expression of a viability-promoting member of the BCL2 family to the induction of hematopoietic cell differentiation. J Biol Chem 1999;274:1801-13.
12. Cavarretta IT, Neuwirt H, Zaki MH, et al. Mcl-1 is regulated by IL-6 and mediates the survival activity of the cytokine in a model of late stage prostate carcinoma. Adv Exp Med Biol 2008;617:547-55.
13. Castro-Rivera E, Samudio I, Safe S. Estrogen regulation of cyclin D1 gene expression in ZR-75 breast cancer cells involves multiple enhancer elements. J Biol Chem 2001;276:30853-61.
14. Bonci D, Coppola V, Musumeci M, et al. The miR-15a-miR-16-1 cluster controls prostate cancer by targeting multiple oncogenic activities. Nat Med 2008;14:1271-7.
15. Kim S, Kang JK, Kim YK, et al. Histone deacetylase inhibitor apicidin induces cyclin E expression through Sp1 sites. Biochem Biophys Res Commun 2006;342:1168-73.
16. Hashimoto Y, Naruyama H, Ando R, Okada S, Tozawa K, Kohri K. [Molecular targeted therapy for prostate cancer]. Hinyokika Kiyo 2008;54:57-61.
17. Honda H, Pazin MJ, Ji H, Wernyj RP, Morin PJ. Crucial roles of Sp1 and epigenetic modifications in the regulation of the CLDN4 promoter in ovarian cancer cells. J Biol Chem 2006;281:21433-44.
18. Landers KA, Samaratunga H, Teng L, et al. Identification of claudin-4 as a marker highly overexpressed in both primary and metastatic prostate cancer. Br J Cancer 2008;99:491-501.
19. Chun JY, Hu Y, Pinder E, Wu J, Li F, Gao AC. Selenium inhibition of survivin expression by preventing Sp1 binding to its promoter. Mol Cancer Ther 2007;6:2572-80.
20. Hansen JB, Fisker N, Westergaard M, et al. SPC3042: a proapoptotic survivin inhibitor. Mol Cancer Ther 2008;7:2736-45.
21. Lee TJ, Jung EM, Lee JT, et al. Mithramycin A sensitizes cancer cells to TRAIL-mediated apoptosis by down-regulation of XIAP gene promoter through Sp1 sites. Mol Cancer Ther 2006;5:2737-46.
22. Seligson DB, Hongo F, Huerta-Yepez S, et al. Expression of X-linked inhibitor of apoptosis protein is a strong predictor of human prostate cancer recurrence. Clin Cancer Res 2007;13:6056-63.
23. Sroka IC, Nagle RB, Bowden GT. Membrane-type 1 matrix metalloproteinase is regulated by sp1 through the differential activation of AKT, JNK, and ERK pathways in human prostate tumor cells. Neoplasia 2007;9:406-17.
24. Duan R, Porter W, Safe S. Estrogen-induced c-fos protooncogene expression in MCF-7 human breast cancer cells: role of estrogen receptor Sp1 complex formation. Endocrinology 1998;139:1981-90.

25. Ouyang X, Jessen WJ, Al-Ahmadie H, et al. Activator protein-1 transcription factors are associated with progression and recurrence of prostate cancer. Cancer Res 2008;68:2132-44.
26. Wei S, Yang J, Lee SL, Kulp SK, Chen CS. PPARgamma-independent antitumor effects of thiazolidinediones. Cancer Lett 2009;276:119-24.
27. Husbeck B, Bhattacharyya RS, Feldman D, Knox SJ. Inhibition of androgen receptor signaling by selenite and methylseleninic acid in prostate cancer cells: two distinct mechanisms of action. Mol Cancer Ther 2006;5:2078-85.
28. Shin T, Sumiyoshi H, Matsuo N, et al. Sp1 and Sp3 transcription factors upregulate the proximal promoter of the human prostate-specific antigen gene in prostate cancer cells. Arch Biochem Biophys 2005;435:291-302.
