# Supplementary material for: Modulation of the Activity of Sp Transcription Factors by Mithramycin Analogues as a New Strategy for Treatment of Metastatic Prostate Cancer
Source: PLoS One. 2012 Apr 19;7(4):e35130. doi: 10.1371/journal.pone.0035130 (PMC3334962; doi:10.1371/journal.pone.0035130)
Supplement: Table S2 — Microarrays data sets downloaded from GEO. (PDF) [file pone.0035130.s006.pdf]

Supplementary Table S2. Microarrays data downloaded from GEO (http://www.ncbi.nlm.nih.gov/geo/)

Tissue	Samples N	GEO DataSet	Platform	References
Normal prostate	18	GDS2545	HG_U95A	4
Primary tumor	65	GDS2545	HG_U95A	4
Metastatic tumor	24	GDS2545	HG_U95A	5
Treated androgen-independent primary tumor	10	GDS1390	HG_U133A	6
Untreated androgen-dependent primary tumor	10	GDS1390	HG_U133A	6
