# Supplementary material for: Modulation of the Activity of Sp Transcription Factors by Mithramycin Analogues as a New Strategy for Treatment of Metastatic Prostate Cancer
Source: PLoS One. 2012 Apr 19;7(4):e35130. doi: 10.1371/journal.pone.0035130 (PMC3334962; doi:10.1371/journal.pone.0035130)
Supplement: Table S3 — PCR primer sets and sequences. (PDF) [file pone.0035130.s007.pdf]

Table S3. PCR primer sets and sequences

Quantitative RT-PCR

Gene name	Accession No	Forward primer	Reverse primer
GAPDH	NM_002046.3	ccatgttcgtcatgggtgt	ccaggggtgctaagcagtt
B2M	NM_004048.2	ttctggcctggaggctatc	tcaggaaatttgactttccattc
VEGFA	NM_001033756.1	agtgtgtgcccactgagga	ggtgaggtttgatccgcata
MYC	NM_002467.3	tgctccatgaggagacacc	cttttccacagaaacaacatcg
C-SRC	NM_198291.1	cgagaaagtgagaccacgaa	ttggcgttgtcgaagtca
hTERT	NM_198255.2	gccttcaagagccacgtc	ccacgaactgtcgcatgt
MCL1	NM_182763.1	aagccaatgggcaggtct	agaactccacaaacccatcct
CCND1	NM_053056.2	gaagatcgtcgccacctg	gacctcctcctcgcacttct
CCNE1	NM_001238.1	cctcggattattgcaccatc	cctctctatttgcccagctc
BIRC5	NM_001168.2	agaactggcccttcttgga	caagtctggctcgttctcagt
XIAP	NM_001167.2	ttttgggacatggatatactcagtt	agcactttactttatcaccttcacc

Quantitative PCR of genomic DNA

Gene name	Gene ID	Forward primer	Reverse primer
ACTB	60	ctgttttgtggcttgttcag	aggaaaccttccctcctcta
B2m	12010	ttggttgagaagcagaaaca	cacacagtcaagttcccaaa

CHIP analysis

Gene name	Gene ID	Forward primer	Reverse primer
VEGFA	7422	gtccgcacgtaacctcactt	ccaaggttcacagcctgaaa
MYC	4609	agggatcgcgctgagtataa	tgcctctcgctggaatttact

RT-PCR

Gene name	Accession No	Forward primer	Reverse primer
GAPDH	NM_002046.3	ccatgttcgtcatgggtgt	ccaggggtgctaagcagtt
SP1	NM_138473.2	ctatagcaaatgccccaggt	tctgggctgttttctccttc
SP3	NM_001172712.1	ttgcacctgtcccaactgta	tgttgcttctttttcccaaga
SP4	NM_003112.3	tcgaagagttgcctgttcct	tcctggttcattactgcctctt
